# Selenium intake and risk of type 2 diabetes among adults in Saudi Arabia

**DOI:** 10.3389/fendo.2025.1579223

**Published:** 2025-05-30

**Authors:** Waad Alfawaz, Maha Alharithy, Sara AL-Musharaf, Madawi Aldhwayan, Ghadeer S. Aljuraiban

**Affiliations:** Department of Community Health Sciences, College of Applied Medical Sciences, King Saud University, Riyadh, Saudi Arabia

**Keywords:** selenium intake, type 2 diabetes, insulin resistance, hyperglycemia, blood glucose

## Abstract

**Introduction:**

Recent studies have demonstrated mixed findings regarding the intake of selenium (Se), an essential trace element for antioxidant defense and insulin metabolism regulation. Some studies have linked high Se intake to a higher risk of type 2 diabetes (T2DM), while others found protective or null associations. Se plays a dual role in that it aids pancreatic b-cell function and reduces oxidative stress, while excessive amounts disrupt redox balance and impair glucose metabolism.

**Methods:**

The present study aimed to investigate the relationship between Se intake and markers of glucose metabolism in a cross-sectional sample of 1074 adults in Saudi Arabia with (n=213) and without (n=861) T2DM as determined by physician diagnosis. Dietary Se intake was assessed using a validated Saudi Food Frequency Questionnaire and analyzed with ESHA software. Sociodemographic, anthropometric, clinical, and lifestyle data were collected. The relationships between Se intake and the presence of a T2DM diagnosis, fasting blood glucose (FBG), fasting insulin, and Homeostatic Model Assessment of Insulin Resistance (HOMA-IR), were assessed using multivariate linear and logistic regression analyses, adjusting for age, sex, education, income, body mass index, physical activity, smoking, medication use, and family history of T2DM.

**Results:**

The average Se intake was lower in adults with T2DM compared to those without (20 mg/1000 kcal and 38 mg/1000 kcal, p<0.0001, respectively). Multivariate linear regression analysis demonstrated no statistically significant association between the Se intake and FBG, HOMA-IR index, and fasting insulin levels after adjustments. Multivariate logistic regression revealed a significant association between dietary Se and a diagnosis of T2DM when fully adjusted [OR 0.8 (95% CI: 0.7, 0.9, P<0.05)].

**Discussion:**

In conclusion, our research found that Saudi adults with relatively higher Se intake had lower odds of T2DM diagnosis, while no significant relationships were found between Se intake and glycemic biomarkers. Future studies incorporating longitudinal data and serum Se levels can further clarify the relationship.

## 1 Introduction

Evidence suggests that Type 2 Diabetes Mellitus (T2DM) is increasing in prevalence worldwide thus enhancing the incidence of related morbidity, mortality, and financial burden (1). Complications of T2DM such as cardiovascular disease (CVD), kidney failure and blindness affect the quality of life and increases the medical costs. At present, 537 million adults between 20–79 years have been diagnosed with T2DM globally. This number is projected to rise to 643 million by 2035 and to 783 million by 2045 (2). Furthermore, other estimates, such as those from the Global Burden of Disease study, predict that the global prevalence of diabetes could reach 1.31 billion by 2050 (3).

Saudi Arabia is not exempt from this crisis, with an estimated seven million people who are diabetic. The country has the second highest prevalence of diabetes in the Middle East and the seventh highest in the world (4). Data from the International Diabetes Federation shows that the age-standardized prevalence of diabetes in Saudi Arabia was approximately 23% in 2024, higher than the global average of 11%, and exceeding regional rates like the United Arab Emirates at 21% and Bahrain at 22% (5). The widespread prevalence of diabetes is further compounded by lifestyle and dietary transitions in the country, such as urbanization (6), adoption of sedentary behavior, and unhealthy eating habits rich in energy-dense yet nutrient-deficient foods (7, 8). Recently, studies have also explored the relationship between specific nutrients and T2DM which have produced significant results (9, 10).

Selenium (Se) is an essential trace element that has been associated with T2DM and glucose metabolism through several mechanisms. As an essential trace element, Se is a component of selenoproteins and is an antioxidant that reduces the reactive oxygen species (11). Lack of Se in this process leads to oxidative stress, beta-cell damage and insulin resistance. However, Se can also cause hyperglycemia and hyperinsulinism as a result of disrupting the body’s redox system. It has been reported that the relationship between Se intake and T2DM risk follows a U-shaped curve, with both low and high levels of Se intake associated with increased risk of T2DM (12).

A meta-analysis of non-experimental studies published in 2021 revealed that the risk of T2DM was increased with higher serum Se concentrations (13). Similarly, a meta-analysis of observational studies in 2019 revealed high serum Se level was significantly associated with high risk of T2DM (14). In contrast, a systematic review of randomized controlled trials (RCTs) in 2023 revealed some benefits of Se on fasting insulin (15), while another systematic review in 2022 of observational studies showed no association between gestational diabetes and Se intake (16).

A review of recent studies following those systematic reviews have also shown contradictory findings. For example, a large study in China reported that Se supplementation at a dose of 52 μg per day increased the risk of T2DM by about 20%. However, the authors noted that population bias may have affected the results, which was considered a major limitation in this study (17). On the other hand, a cohort study that followed a Chinese sample population of 14,711 individuals for 9.2 years did not find any association between dietary Se and the incidence of T2DM (18). Furthermore, a cross-sectional study in Brazil showed no association between high Se intake of about 157 µg/day and the prevalence of T2DM (19).

One of the challenges in understanding the relationship between Se and T2DM is that Se intake can vary greatly within regions and even within the same country due to variation in soil Se content (20). Given these complexities, it is important that country-specific studies be carried out in order to determine how Se intake may be related to T2DM risk. The current study aims to investigate the relationship between Se intake and markers of glucose metabolism to understand how Se is linked to glucose regulation in adults in Saudi Arabia.

## 2 Materials and methods

### 2.1 Study design

This is a cross-sectional study investigating the relationship between Se intakes and T2DM and glucose metabolism biomarkers in adults in Saudi Arabia. The local institutional ethics committee of King Saud University approved the study (No. 22/0319/IRB).

### 2.2 Participants

**Figure 1:**
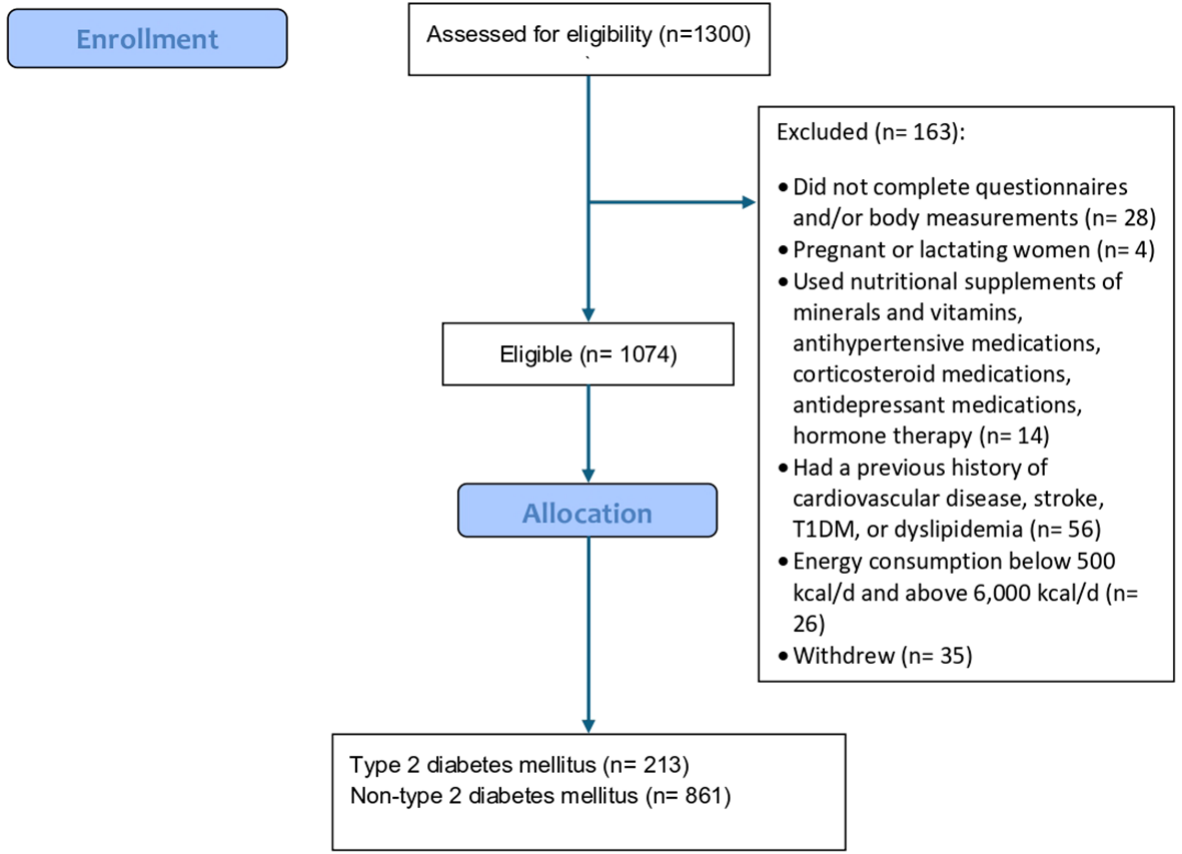
Flow diagram.

Adults from the general population were invited by social media platforms to participate between December 2021 and December 2023. Inclusion criteria were: individuals aged ≥18 years, residing in Riyadh city during the study period, and able to provide informed consent. At the Nutrition Clinic in King Saud university, those who agreed to participate signed a consent form after being briefed about the study and data that needed to be collected. Participants were informed that they had the right to withdraw from the study at any time. A total of 1300 people were recruited, 1074 of them completed the study, and 163 were excluded because they were pregnant or lactating women, used nutritional supplements of minerals and vitamins, antihypertensive medications, corticosteroid medications, antidepressant medications, hormone therapy, and had a previous history of CVD, stroke, T1DM, or dyslipidemia. Furthermore, those with energy intake below 500 kcal/d and above 6,000 kcal/d were also excluded (see flow diagram, **Figure 1**).

### 2.3 Data collection

Data were collected using various methods, including questionnaires, laboratory testing, and anthropometric measurements. Trained dietitians conducted interviews with each participant, collecting information on their sociodemographic characteristics, health history, and dietary habits on the same day as the recruitment. Afterwards, appointments were scheduled for the collection of blood samples. All biochemical analyses were performed during a single lab visit, and participants were provided with their results.

### 2.4 Sociodemographic data

Through structured questionnaires, we obtained information on sociodemographic and lifestyle variables. Sociodemographic data collected included age (continuous), sex, education level (primary, intermediate, high school, diploma degree, bachelor’s degree, postgraduate), and income (classified according to the standards defined by the Saudi General Authority for Statistics). Self-reported smoking status (yes/no) was also collected as part of the questionnaire.

We assessed physical activity using the Arabic version of the Global Physical Activity Questionnaire (GPAQ) (21). GPAQ is categorized into four categories: occupational physical activity, transport-related physical activity, physical activity during discretionary or leisure time, and sedentary behavior. GPAQ incorporates physical activity elements such as intensity, duration, and frequency based on the total Metabolic Equivalent of Task (MET) minutes per week. Physical activity was categorized as high (engaging in vigorous-intensity activity on at least 3 days, accumulating a minimum of 1500 MET minutes per week; or 7 or more days of any combination of walking, moderate- or vigorous-intensity activities, achieving at least 3,000 MET minutes per week), moderate (participating in 5 or more days of moderate-intensity activity or walking, totaling at least 600 MET minutes per week; or 5 or more days of any combination of walking, moderate- or vigorous-intensity activities, achieving a minimum of 600 MET minutes per week), or low (not meeting the criteria for either high or moderate levels of physical activity).

### 2.5 Dietary data

Dietary Se intake was evaluated using the Saudi Food Frequency Questionnaire (FFQ) (22). The Saudi FFQ was created based on the United States (US) semi-quantitative FFQ (23), translated into Arabic by the Saudi Food and Drug Administration, and modified to include foods and beverages commonly consumed in traditional Saudi cuisine. The Saudi FFQ includes a list of 133 items, and participants are asked to recall the frequency with which they consumed each item over the past year.

Furthermore, to evaluate the validity of the FFQ, two non-consecutive 24-hour dietary recalls were also collected from all participants (24). The first recall was collected on the recruitment day, and the second by phone two weeks later. The average Se intake from the 24-hour dietary recalls was compared with the intake reported by the FFQ. Pearson correlation coefficients showed a strong positive correlation between Se intake estimated by the FFQ and the average intake from the recalls (r = 0.68, p < 0.001), indicating good relative validity of the FFQ for assessing habitual Se intake.

Due to the absence of a comprehensive national food composition database for Saudi Arabia, the US Department of Agriculture (USDA) Food and Nutrient Database was used as the most practical and regionally applicable source available. The Se content of traditional Saudi foods and beverages in the FFQ was determined either through matching each food item to its USDA Food and Nutrient Database counterpart or by disaggregating complex dishes into their main ingredients. Each ingredient was then matched to a corresponding entry in the USDA database, and the Se content was calculated based on standard recipes and portion sizes (25). This approach enabled us to achieve the most accurate Se content estimation possible despite the absence of a national food composition table for Saudi Arabia. We analyzed the FFQ using the Elizabeth Stewart Hands and Associates (ESHA) food analysis software (version 11.1) and the USDA Food and Nutrient Database. All sources of Se intake were combined and standardized per 1000 kcal of energy.

### 2.6 Anthropometric measurements

The anthropometric measurements, including body weight and height were taken by a trained dietitian. The average of two measurements was used for the final analysis. Weight and height were assessed without heavy clothes and shoes. Weight was obtained using Bioelectrical Impedance Analysis (BIA) (Body Composition Analyzer; In Body 570, Seoul, South Korea). Height was measured using a digital stadiometer (Seca 274, Hamburg, Germany). Body mass index (BMI) was calculated as weight in kilograms divided by the square of height in meters (*kg*/*m* ^2^).

Medical history was collected including self-reported family history of T2DM (yes/no) and a prior T2DM diagnosis (yes/no). Participants were divided into those with T2DM (n=213) and those without T2DM (n=861). Participants with T2DM were defined as those who reported being diagnosed with T2DM by a physician and showed proof of diagnosis, such as medical records or a prescription.

Blood samples were collected from participants at the labs of King Saud University. Fasting blood glucose (FBG) levels (8–10 hours) were measured using citrate-based tubes, which are recognized for their effectiveness in immediately inhibiting glycolysis, thereby ensuring the accuracy and reliability of glucose measurements. Plasma and serum were collected by centrifuging the whole blood at 1,200 x g for 15 minutes and were immediately stored at -80°C. Fasting plasma insulin levels were measured using an Enzyme-Linked Immunosorbent Assay (ELISA), which provides high sensitivity and specificity for insulin quantification. Homeostatic Model Assessment of Insulin Resistance (HOMA-IR) index was calculated by multiplying the fasting plasma insulin level (measured in milliunits per liter) by FBG (measured in millimoles per liter), and then dividing the result by 22.5 (26).

### 2.7 Statistical analysis

Continuous and categorical data were expressed in terms of mean ± standard deviation (SD) and percentage, respectively. For skewed variables, we also reported medians and interquartile ranges (IQRs). The normality of all continuous variables was assessed using the Shapiro-Wilk test, and showed that the main variables of interest (e.g., FBG) did not significantly deviate from normal distribution. Accordingly, independent sample t-test was used to compare participant characteristics between those with and without T2DM. We used non-parametric tests like the Mann–Whitney U test whenever non-normal distribution patterns emerged.

Prior to regression analysis, we applied Spearman’s rank correlation coefficient to examine associations with FBG, insulin resistance (HOMA-IR), and fasting insulin levels. A correlation was considered statistically significant at p < 0.05. Multivariate linear regression was used to assess the association between the dietary Se and FBG, HOMA-IR index, and fasting insulin levels, while logistic regression was used to examine the association between the presence of a T2DM diagnosis and Se intake. For both regression analyses, Model 1 was adjusted for age and sex. Model 2 was adjusted for age, sex, education, income, BMI, physical activity, smoking, use of medication, and family history of T2DM. These adjustments were literature-driven, as key systematic reviews and meta-analyses have shown how factors like education, income, occupation, and health behaviors influence both Se intake and T2DM (27, 28). Effect modification by T2DM status was tested by including interaction terms between T2DM status and dietary Se intake in the multivariate linear regression models assessing associations with glucose metabolism markers (FBG, HOMA-IR, and fasting insulin). No statistically significant interactions were observed. All data were analyzed using Statistical Analysis System^®^ (SAS 9.3) software. A p-value <0.05 was considered statistically significant, and the estimate’s precision is indicated by a 95% confidence interval (CI).

## 3 Results

A total of 1074 participants were included in the analysis, comprising 32% males and 68% females, with a mean age of 32.7 ± SD:11.1 years. About half (i.e., 54.8%) of the participants had a bachelor’s degree, and around a third (39.7%) had a monthly income between SAR 10,000 and 20,000 (**Table 1**).

**Table 1:** Characteristics of the study population, overall and stratified by type-2 diabetes diagnosis (n = 1074)^a^.

Characteristics	Total N=1074	Without T2DM N=861	With T2DM N=213	P-value
Age at recruitment (years)	32.7 ± 11.1	31.7 ± 10.5	32.9 ± 11.2	0.81
Sex (%)				
Males	32	26.8	32.8	0.44
Females	67.9	73.1	67.1	
Education (%)				
Primary	1.2	0	1.4	0.32
Intermediate	1.6	2.4	1.4	
High school	18.9	29.2	17.3	
Diploma degree	8.9	2.4	9.9	
Bachelor’s degree	54.8	51.2	55.3	
Postgraduate	14.4	14.6	14.3	
Monthly family income (%)				
Less than 5000 SR	7.6	4.8	8.1	0.11
Between 5000 to 10000 SR	23.7	17	24.7	
Between 10000 to 20000 SR	39.7	48.7	38.3	
Above 20000 SR	26.2	21.9	26.9	
Other	0.6	0	0.7	
Smoker (%)				
No	90	87.8	90.4	0.6
Yes	9.94	12.2	9.5	
Physical activity (%)				
Low activity	93.27	100	92.2	0.06
High activity	6.73	0	7.7	
Family history of diabetes (%)				
No	14.6	90	25	<0.0001
Yes	85.4	10	75	
Use of insulin therapy (%)*				
No	98	100	97.7	0.33
Yes	1.9	0	2.2	
BMI (Kg/m^2^)	27.2 ± 6.2	27.3 ± 6.9	27.2 ± 6.1	0.26
Average energy intake (kcal/day)	3635 (1301)	3905 (850)	3249 (1670)	0.07
Dietary Se intake (μg/1000 kcal)	32 (16)	38 (18)	20 (12)	<0.0001
FBG (mg/dl)	94.0 (15.7)	90.9 (13.1)	96.6 ± 16.2	0.07
HOMA-IR †	2.1 (1.6)	1.9 (2.1)	2.4 (2.6)	<0.0002
Fasting insulin level (uIU/mL) †	9.0 (8.1)	7.5 (5.6)	10.9 (9.7)	0.05

^a^Values are presented as mean ± standard deviation,or median (interquartile ranges) where appropriate),or %.

BMI, body mass index; HbA1c, hemoglobin A1C; FBG, fasting plasma glucose; HDL, high density lipoprotein; HOMA-IR, homeostasis model assessment of insulin resistance; LDL, low density lipoprotein; NC, neck circumference; Se, selenium; TG, triglycerides; WC, waist circumference and WHR, waist-to-hip ratio.

P-values are for test of difference between group without T2DM and group with T2DM determined by t-test.

*Medication use reflects insulin therapy only. Most participants with T2DM were using oral hypoglycemic agents or managed the condition through lifestyle changes.

†Median (IQR).

The prevalence of T2DM in the study population was 19.8%. When comparing the baseline characteristics, family history of T2DM diagnosis (75.0% vs 10.0%, p<0.0001), HOMA-IR [2.4 (IQR 2.6) vs. 1.9 (IQR 2.1), p<0.0002], and fasting insulin [10.9 (IQR 9.7)] vs 7.5 (IQR 5.6) uIU/mL, p=0.05] were significantly higher among those with T2DM compared to those without. The median dietary Se intake was 20 (IQR=12) µg/1000 kcal, significantly lower in those with compared to those without T2DM [38 (IQR=18) µg/1000 kcal, p <0.000] (**Table 1**).

The correlation between dietary Se intake and FBG levels was significant (r= 0.20, p<0.05). However, no significant correlation was observed between Se intake and either HOMA-IR or fasting insulin levels (**Table 2**). In the multivariable linear regression analysis, after adjusting for age, sex, BMI, physical activity, and smoking status (model 2), Se intake was positively associated with FBG [0.02 (-0.11, 0.16), p > 0.05], but was not statistically significant. Similarly, Se intake was associated with higher HOMA-IR levels [(0.02 (-0.33, 0.37), p > 0.05] (**Table 3**).

**Table 2:** Spearman correlation between dietary selenium intake and fasting blood glucose, insulin resistance (HOMA-IR), and fasting insulin levels in Saudi adults (n=1074)**^a^**.

Variable	FBG (mg/dl)	HOMA-IR	Fasting insulin level
Dietary Se intake	0.20	0.02	0.03

^a^Correlations are statistically significant,except those ranging from –0.03 to 0.03.

**Table 3:** Mean difference in fasting blood glucose, insulin resistance (HOMA-IR), and fasting insulin levels per 1-unit increase in dietary selenium intake (μg/1000 kcal) in Saudi adults (n=1074)^a^.

Variable	Fasting blood sugar (mg/dl)	P value	HOMA-IR	P value	Fasting insulin level	P value
	Mean difference (95%CI)		Mean difference (95%CI)		Mean difference (95%CI)	
Dietary selenium (μg/1000 kcal)						
Model 1	0.01 (-0.12, 0.15)	0.23	0.02 (-0.32, 0.36)	0.30	-0.01 (-0.07, 0.07)	0.20
Model 2	0.02 (-0.11, 0.16)	0.25	0.02 (-0.33, 0.37)	0.35	-0.01 (-0.08, 0.07)	0.22

^a^Values are presented as mean (95% CI,confidence intervals).

Model 1: Adjusted for age and gender.

Model 2: Adjusted for model 1+ education,income,body mass index,physical activity,smoking,use of insulin therapy,family history of diabetes.

Logistic regression analysis further revealed a significant association between dietary Se and T2DM (OR = 0.7 (95% CI: 0.7, 0.9, p = 0.03). This association persisted even after full adjustment for confounding variables (Model 2 OR = 0.8 (95% CI: 0.7, 0.9), p = 0.04) (**Table 4**).

**Table 4:** Adjusted odds ratios (95% CI) for type 2 diabetes per 1-unit dietary selenium intake (μg/1000 kcal) in Saudi adults (n=1074)^a^.

Variable	Cases/N	Model 1				Model 2			
		OR	Lower 95% CI	Upper 95% CI	P value	OR	Lower 95% CI	Upper 95% CI	P value
Diabetes ^a^	213/861								
Dietary selenium (μg/1000 kcal)	17.3 ± 18.5	0.7	0.7	0.9	0.03	0.8	0.7	0.9	0.04

^a^Values are presented as odds ratio (95% CI,confidence intervals).

Model 1: Adjusted for age and gender.

Model 2: Adjusted for model 1+ education,income,body mass index,physical activity,smoking,use of insulin therapy,family history of diabetes.

## 4 Discussion

### 4.1 Interpretation of findings

This is the first cross-sectional study conducted to investigate the relationship between dietary Se intake and T2DM, FBG, HOMA-IR, and fasting insulin in Saudi Arabia. We found that in participants without T2DM, the median dietary Se was 112 µg/day (data not shown), which is higher than the recommended total daily intake of 55–70 µg/day for adults, and higher relative to the intake observed in those with T2DM 65 µg/day (29). Higher dietary selenium intake was significantly associated with reduced odds of T2DM (OR = 0.7), and this association remained robust after adjusting for confounders (Model 2 OR = 0.8). However, there was no association between dietary Se and biomarkers of glucose metabolism in linear regression models.

### 4.2 Comparison with previous studies

Previous research supports the inverse relation between Se intake and T2DM. For example, an RCT conducted in Greece among middle-aged adults with T2DM showed that daily supplementation of 200 μg/d of Se significantly reduced glycemic profiles including FBG, postprandial glucose, and insulin resistance markers (e.g., HOMA-IR) (30). Another study showed that higher Se intake was associated with lower insulin resistance in 2,420 non-diabetic participants (31). However, other studies report contradictory results. However, the study conducted in China showed a positive correlation between dietary Se intake and the incidence of T2DM (17). Another cross-sectional study that assessed Se intake and T2DM among 4,106 participants showed no association between Se intake and the prevalence of T2DM (19). Furthermore, a cross-sectional study conducted among 290 women, including 140 T2DM patients and 150 controls, revealed a significant association between the risk of T2DM and both high and low dietary Se intake (32). Regarding plasma levels, a prospective cohort study conducted on 1,039 incident cases of T2DM and 1,039 controls showed that the fasting plasma samples of Se was positively associated with incident T2DM (32).

These inconsistencies likely originate from several factors, including differences in study design, Se sources used (dietary vs. supplemental), baseline Se status of participants, or specific factors unique to each population. For example, people in the US typically have higher Se levels due to the soil and diet, which may lead to Se intakes that exceed the threshold for metabolic benefits. In contrast, participants in our study may have had lower baseline levels of Se. Furthermore, the type of study affects the interpretation of results. RCTs that focus on supplements reveal different outcomes compared to observational studies assessing habitual dietary intake. In addition, factors like genetics, lifestyle, and environment may also influence Se metabolism and its effects on glucose homeostasis.

### 4.3 Biological plausibility

The relationship between Se intakes and T2DM is supported with biological mechanisms. For instance, a high level of the Se enzyme, specifically glutathione peroxidase, improves β-cells’ function and protects them from oxidative stress (33). Moreover, selenite treatment enhances adipocyte differentiation and function, as well as insulin receptor expression, by boosting selenoprotein glutathione peroxidase (GPx3) expression (34). Elevated GPx3 expression can decrease oxidative stress in adipose tissue, improve insulin signaling pathways, glucose uptake, and sensitivity. The reduction in oxidative stress can also decreases chronic low-grade inflammation, which is a major factor in insulin resistance. Collectively, these processes contribute to improved glycemic control, as shown by lower FBG levels and improved insulin response, leading to reduced risk of developing T2DM (34).

Despite this mechanism, the association of Se intake with T2DM remains contradictory. Our study found no statistically significant relationship between dietary Se intake and FBG, HOMA-IR, or insulin. The absence of a link between Se consumption and glycemic biomarkers implies Se’s metabolic function is context-dependent, influenced by initial Se levels and the type of Se consumed along with unidentified factors. Previous studies reported that the effects of Se show complexity because they follow a U-shaped curve where both low levels and high levels can cause harm (13). This suggests that if Se has any role in glucose metabolism, it was not detectable using the selected biomarkers in this study.

While our findings did not reveal a significant association between dietary Se and glycemic markers, it is important to consider regional studies. A study in Saudi Arabia has shown that the average Se intake is approximately 93 μg/day, with the main sources being meat, eggs, and cereals (35). Similar to our study, this intake is higher than global recommended range of 55–70 μg/day (29), which suggests higher Se intake among the Saudi population. However, it is worth noting that data linking Se intake to T2DM prevalence is limited in Saudi Arabia, highlighting the need for further localized research.

### 4.4 Study strengths and limitations

The study has several strengths. We used a detailed FFQ specifically for the Saudi population and extensive sociodemographic and dietary information to identify confounding variables, and validated the FFQ using two 24-hour dietary recalls. To account for differing energy intakes and requirements, we standardized the Se intakes per 1000 kcals. However, there are a number of limitations, including the cross-sectional design, which precludes causal inferences and judgments about temporality in the relationship between Se and T2DM. The diagnosis of T2DM and dietary intakes were obtained from self-reported data and thus are subject to recall bias despite active steps taken to limit this. We relied on dietary intakes rather than serum Se level. Dietary intakes are not necessarily reflective of the bioavailability of Se. The bioavailability and absorption can be influenced by several factors, including genetics (e.g., polymorphisms in selenoprotein genes), the food matrix (e.g., Se in animal-based foods is generally more bioavailable than in plant-based sources), the chemical form of Se (e.g., selenomethionine vs. selenite), and interactions with other dietary components (e.g., high fiber, phytates, or heavy metals such as mercury can reduce Se absorption). The food composition database used to assess intakes would not account for the variations in Se that occur due to soil differences, food processing methods, (e.g., milling or refining grains reduces Se content), cooking methods (e.g., boiling can leach Se from foods), and storage conditions (e.g., prolonged storage can degrade Se content in certain foods). Another limitation is the dependence on the USDA Food and Nutrient Database for Se values due to the absence of a comprehensive national food composition table for Saudi Arabia. Despite its widespread regional application, the use of the USDA Food and Nutrient Database may not accurately reflect the Se content of traditional Saudi dishes, introducing potential misclassification in nutrient estimates. Furthermore, using food data with aggregated or averaged Se contents may fail to capture the true variability in individual intakes. This can lead to potentially underestimating extremes and biasing results toward the mean, weakening the observed associations and potentially explaining the non-significant findings.

All study participants were Saudi adults; therefore, our findings cannot be generalized to other ethnic groups or people of varying ages. The participants in this study were however reflective of the general Saudi population in terms of key demographics, including income and education levels, based on national survey comparisons. Since Se intakes are often regionally specific, even within a country, the generalizability may also be limited within Saudi Arabia except to those with comparable dietary intakes. Participants were recruited from Riyadh (the Central Region), which should be considered when interpreting the findings. Finally, residual confounding and information bias are possible.

## 5 Conclusions

In this cross-sectional study, dietary Se intake was lower among individuals with T2DM compared to those without T2DM. However, Se intake was not significantly associated with FBG, insulin resistance, or fasting insulin. Future studies incorporating longitudinal data and serum Se levels would be valuable for examining associations with insulin resistance and glycemic control, including HbA1c, in the Saudi population. Experimental studies can be used to identify the optimal range of Se intake intended to minimize the risk of T2DM.

## Data Availability

The raw data supporting the conclusions of this article will be made available by the authors, without undue reservation.
